# iRGD: A Promising Peptide for Cancer Imaging and a Potential Therapeutic Agent for Various Cancers

**DOI:** 10.1155/2019/9367845

**Published:** 2019-06-26

**Authors:** Houdong Zuo

**Affiliations:** Sichuan Key Laboratory of Medical Imaging, Department of Radiology, Affiliated Hospital of North Sichuan Medical College, Nanchong, Sichuan 637000, China

## Abstract

Poor penetration into the tumor parenchyma and the reduced therapeutic efficacy of anticancer drugs and other medications are the major problems in tumor treatment. A new tumor-homing and penetrating peptide, iRGD (CRGDK/RGPD/EC), can be effectively used to combine and deliver imaging agents or anticancer drugs into tumors. The different “vascular zip codes” expressed in different tissues can serve as targets for docking-based (synaptic) delivery of diagnostic and therapeutic molecules. *α*v-Integrins are abundantly expressed in the tumor vasculature, where they are recognized by peptides containing the RGD integrin recognition motif. The iRGD peptide follows a multistep tumor-targeting process: First, it is proteolytically cleaved to generate the CRGDK fragment by binding to the surface of cells expressing *α*v integrins (*α*v*β*3 and *α*v*β*5). Then, the fragment binds to neuropilin-1 and penetrates the tumor parenchyma more deeply. Compared with conventional RGD peptides, the affinity of iRGD for *α*v integrins is in the mid to low nanomolar range, and the CRGDK fragment has a stronger affinity for neuropilin-1 than that for *α*v integrins because of the C-terminal exposure of a conditional C-end Rule (CendR) motif (R/KXXR/K), whose receptor proved to be neuropilin-1. Consequently, these advantages facilitate the transfer of CRGDK fragments from integrins to neuropilin-1 and consequently deeper penetration into the tumor. Due to its specific binding and strong affinity, the iRGD peptide can deliver imaging agents and anticancer drugs into tumors effectively and deeply, which is useful in detecting the tumor, blocking tumor growth, and inhibiting tumor metastasis. This review aims to focus on the role of iRGD in the imaging and treatment of various cancers.

## 1. Introduction

Cancer is a prominent disease and a leading cause of mortality worldwide [1]. In 2018, 1,735,350 new cancer cases and 609,640 cancer-related deaths were estimated to occur in the United States. Lung and breast cancers are the most frequently diagnosed cancers and the leading causes of cancer-related death in men and women, respectively [1]. Early detection is important for cancer therapy. Currently, magnetic resonance imaging (MRI) and ultrasound (US) are the most reasonable imaging modalities used for the early detection and evaluation of cancer after preoperative chemotherapy or surgery [2]. Cancer imaging is crucial for early detection; therefore, the development of cancer imaging agents that can be effectively delivered to tumor parenchyma is a research hotspot.

The current main therapeutic options for most cancers, such as breast and gastric cancer, are surgical resection and clinical chemotherapy [3, 4]. However, the current challenge of systemic therapy is poor penetration into the tumor parenchyma and the adverse effects of the drugs. In some solid tumors, many drugs can only penetrate 3-5 cell diameters from the blood vessels into the parenchyma and exhibit fewer anticancer effects [5]. The vascular zip codes, which are representative of biochemical signatures in different tumor tissues, may serve as potential targets for docking-based diagnostic and therapeutic delivery [6, 7]. Therefore, cancer-targeted therapies are necessary and significant. The *α*v integrins (*α*v*β*3 and *α*v*β*5) are highly expressed in the cancer vasculature and can bind peptides containing the RGD integrin recognition motif  [7]. RGD peptides are a kind of peptide that contain Arg-Gly-Asp sequence, which is widely found in the living body and is the recognition site for integrins and its ligand protein interaction. In tumor blood vessels, two key molecules (neuropilin-1, NRP-1, and neuropilin-2, NRP-2) can regulate tumor tissue vasculature permeability and enhance permeability through the interaction between the C-end Rule (CendR) motif and neuropilin [8]. NRP involved as a coreceptor of key receptor tyrosine kinases (RTKs) in many signaling pathways is of particular importance for cell survival and cell fate. NRP1 is a 120 kDa and NRP2 is a 112 kDa transmembrane glycoprotein [9]. NRP1 was widely found in neuronal cells and blood vessels of different tissues, especially in arterial vessels, and NRP2 is expressed in neural crest-derived cells [10]. Integrins have been reported to interact with NRPs, because NRPs are versatile in their structure and repertoire of soluble ligands [11]. The interaction between the CendR motif and NRP-1 seems to be a vital factor for penetration of biological barriers. And the R/KXXR/K motif is considered to be as a mediator of cell and tissue penetration [12]. Therefore, the strict requirement for the CendR motif is to be at the C-terminus of the peptide to facilitate cell binding. This motif cannot be activated unless it occupies a C-terminal position in the peptide and this position effect is named the C-end Rule (CendR). In general, the typical characteristics of CendR are as follows: (1) R/KXXR/K recognition motif; (2) requirement for C-terminal exposure of the motif for activity; (3) transition of internal CendR motifs to active, C-terminal ones through proteolytic cleavage; and (4) NRP-1 dependence of the recognition and penetration activities [12].

iRGD is a newly identified peptide. Similar to conventional RGD, iRGD can bind to the surface of cancer cells with high levels of *α*v integrin expression [13]. iRGD plays a role following a multistep process.

The CRGDK fragment is generated when iRGD is injected intravenously and functions by targeting *α*v integrins and exposing the CendR motif at the C-terminus to activate the RXXR/K sequence motif. The interaction between the CendR motif and NRPs activates the bulk delivery process, allowing anticancer drugs to penetrate deeply into the tumor tissue through conjugation to or even coadministration with iRGD [14, 15]. Therefore, iRGD can serve as an effective therapeutic target to enhance the efficacy of anticancer drugs. This review aims to highlight the role of iRGD peptide in cancer imaging and therapy.

## 2. iRGD Peptide

Conventional RGD binds specifically to integrin *α*v*β*3 on the surface of the tumor vasculature [16] and plays an important role in delivering anticancer drugs, imaging agents, nanoparticles, and virus vectors to blood vessels [13, 14, 17]. The iRGD peptide, with its short amino acid sequence (Arg-Gly-Asp), is a recently identified type of tumor-targeting peptide that was discovered by phage display. This peptide can control the permeability of tumor cells, regulate cellular internalization and extravasation, and promote deeper tissue penetration to improve the imaging sensitivity and therapeutic efficacy [13, 14]. Like the conventional RGD peptide, iRGD (CRGDK/RGPD/EC) can bind to *α*v integrins that are specifically expressed on the surface of tumor vessels when intravenously injected first [13, 18]. Then, iRGD is cleaved into CRGDK/R by a protease. Because iRGD has a functional sequence, it has affinity for NRP-1, specifically for the active C-end Rule (CendR) motif (R/KXXR/K) exposed at the C-terminus [12]. Tumor tissue penetration is triggered by peptide binding to integrins through the interaction of the CendR motif with NRP-1. This binding process allows the extravasation and penetration of imaging agents or drugs either conjugated to the iRGD peptide or coadministered with iRGD into the target tumor tissues and cells [13, 14, 19].

In general, compared with RGD, the iRGD peptide has the following advantages: First, the affinity of iRGD for *α*v integrins is in the mid to low nanomolar range. Second, the proteolytically processed CRGDK fragment gains a stronger affinity for neuropilin-1 than its residual affinity for *α*v integrins because of the C-terminal exposure of the conditional C-end Rule (CendR) motif (R/KXXR/K), whose receptor proved to be neuropilin-1. The above changes facilitate the shift of CRGDK from integrins to neuropilin-1 and the consequent penetration activities. Every phase involved in this multistep process increases the tumor specificity of iRGD conspicuously. Third, iRGD has greater homing biological characteristics possibly owing to the RGD-directed specific homing of the intact peptide [13]. Fourth, the recruitment of iRGD to the cell surface through the iRGD-integrin interaction is likely necessary for the proteolytic cleavage that activates the resulting tumor penetration because protease inhibitors are usually inanimate on cell surfaces but block proteolysis elsewhere [20]. Fifth, the cell penetrating capability of iRGD is far better than that of conventional RGD peptides, possibly because integrins shuttle between the cell surface and intracellular compartments, and some viral pathogens enter into cells utilizing this mechanism [21]. Finally, iRGD has no adverse effect or cytotoxicity on healthy cells [22], which has caused it to gain considerable attention in targeted research.

## 3. The Process of iRGD Peptide Penetration into Tumors

**Figure 1:**
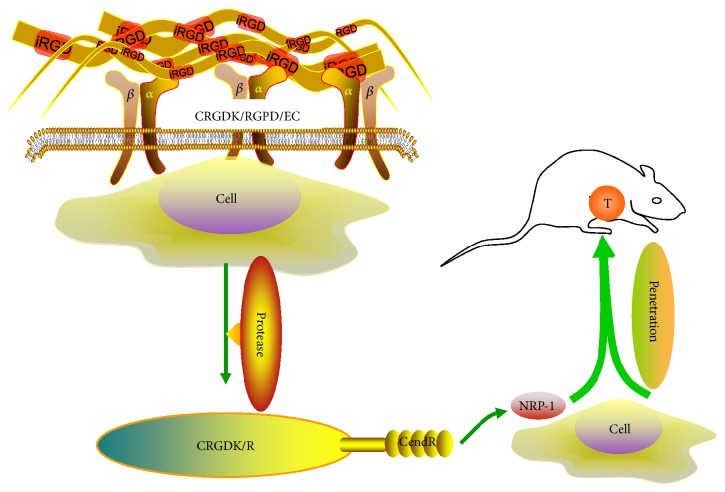
The key steps in the tumor-targeting process of the iRGD peptide. First, iRGD peptides (CRGDK/RGPD/EC) bind to integrins (*α*v*β*3 and *α*v*β*5). Second, the peptide is proteolytically cleaved in the tumor to produce CRGDK/R and expose the activated CendR motif (R/KXXR/K) at the C-terminal end. Third, the CendR motif binds to NRP-1 to trigger tumor tissue penetration. T: tumor.

The phage peptide was first identified by Pasqualini and Ruoslahti in 1996 when a large number of peptides homed to tumor cells were developed [23]. Pasqualini and Ruoslahti discovered new organ-targeting peptide sequences by in vivo screening. They found that some peptides had the ability to regulate the selective localization of phage to brain and kidney vasculature, displaying high selectivity. Then, they synthesized one peptide (CLSSRLDAC) expressed by the brain-localizing phage and demonstrated specific inhibition of homologous phage localization. When the red blood cells were coated with this peptide and injected intravenously, they showed selective localization to the brain. These peptide sequences were first identified targeting endothelial cells, which could be useful in delivering cells, drugs, and genes into the selected tissues in the future [23]. iRGD contains an Arg-Gly-Asp sequence, which can bind to integrins (*α*v*β*3 and *α*v*β*5) specifically expressed on the surface of tumor cells. Briefly, iRGD exerts its function following 3 key steps: (1) iRGD peptides bind to integrins (*α*v*β*3 and *α*v*β*5); (2) the peptide is proteolytically cleaved in tumors to produce CRGDK/R and expose the activated CendR motif (R/KXXR/K) at the C-terminus; and (3) CendR binds to NRP-1 to trigger tumor tissue penetration (Figure 1).

## 4. iRGD for Tumor Imaging

Many nanoparticles, imaging agents, compounds, and ligands coupled, conjugated, and modified by iRGD have been widely studied for tumor imaging and detection [13, 17, 24]. Sugahara et al. [13] intravenously injected iRGD peptide linked to superparamagnetic iron oxide (SPIO) nanoworms into mice with 22Rv1 orthotopic xenograft tumors. The iron oxide nanoparticles showed hypointensity on T2-weighted MR images. The vasculature was hypointense, and the iRGD nanoworms elicited hypointense signal regions in the tumor; by contrast, the CRGDC nanoworms lowered only the signal intensity of the tumor vasculature. These findings were confirmed by optical imaging, which indicated that iRGD was more efficient than conventional RGD peptides [13, 17, 25]. Zuo et al. [22] reported that the iRGD peptide enhanced the positive labeling rate of pancreatic cancer cells and cell uptake with superparamagnetic iron oxide (SPIO) and demonstrated the optimal imaging effect with appropriate concentrations. Moncelet et al. [26] found that either E-[c(RGDfK)2] or iRGD internalization increased the signal-to-noise ratio (SNR), cell labeling, and intracellular signals and improved the MRI diagnosis in glioblastoma cells. In 2016, Xin et al. [27] developed a new tumor-targeting MRI contrast agent by conjugating gadolinium-diethylene triamine pentaacetate (Gd-DTPA) to the bispecific recombinant anti-EGFR-iRGD protein (anti-EGFR-iRGD-DTPA-Gd). This new agent had no obvious cytotoxicity to human gastric carcinoma cells and demonstrated higher T1 relaxivity and superior tumor-targeting ability compared with those of Magnevist. The same findings were also observed and verified by in vivo experiments. Ye et al. [28] artificially synthesized two new iRGD peptide analogues (Ac-Cys(IRDye®800CW)-iRGD and DOTA-Cys(IRDye®800CW)-iRGD) and revealed that both analogues showed significant tumor localization assessed with optical imaging in MDA-MB-435 tumor-bearing mice. Cho et al. [29] synthesized a fluorescence-activatable multifunctional monolithic probe based on a cyclic iRGD peptide for tumor imaging, and it remarkably improved the imaging effect with fluorescence contrast between tumor and normal tissues because the background minimized the tumor-specific signal. Yang et al. [30] synthesized an active-tumor-targeting imaging system with a physical method that used a fabricated iRGD to redshift emissive carbon nanodots (iRGD-CDs). iRGD-CDs showed a higher accumulation in 4T1 cells in vitro; in vivo, iRGD-CDs could penetrate and selectively accumulate in tumors, leading to a better tumor imaging efficacy. The following characteristics may account for the better tumor imaging efficacy: (1) iRGD-CDs have a uniform diameter of 3.3 ± 0.57 nm, well-resolved lattice structures with a d spacing value of 0.22 nm, and longer excitation and emission; (2) iRGD-CDs are stable and do not exhibit obvious attenuation of fluorescence intensity with increases in incubation time; (3) iRGD-CDs have good hemocompatibility and low cytotoxicity; (4) iRGD-CDs could significantly improve the tumor targeting and sensitivity of CDs and could be efficiently delivered to 4T1 tumor tissue; and (5) the iRGD-CD distribution in tumors is much higher than that of the CD group. Przysiecka et al. [31] synthesized CuInZn_x_S_2+x_ quantum dots (QD) electrostatically associated with iRGD (iRGD/QD) to investigate the role of iRGD in the transport of nanoparticles to various human cancer cell lines. Their findings revealed the high penetration ability for the iRGD/QD assembly. Imaging experiments showed that iRGD/QD assemblies were distributed evenly throughout the whole HeLa spheroid, and iRGD/QD may serve as a great potential tumor-targeting imaging agent and/or nanocarrier. A new transformable integrin-targeted ultrasound contrast agent, iRGD microbubbles (iRGD-MBs), which incorporate iRGD-lipopeptides, was developed for tumor angiogenesis imaging. iRGD-MBs showed stronger binding specificity towards endothelial cells than the control agent in vitro. In vivo, iRGD-MBs also displayed stronger enhancement within tumors after intravenous injection in mice bearing 4T1 breast tumors [32]. A novel dual-targeted ultrasound contrast agent (iRGD/CCR2 dual-targeted cationic microbubbles, MB_iRGD/CCR2_) was newly prepared by Xu et al. [33]. The data showed that MB_iRGD/CCR2_ had higher binding efficacy with bEnd.3 cells and MCF-7 cells, as well as loading pGPU6/GFP/Neo-shAKT2 plasmid DNA more effectively, and had higher gene transfection efficiency under ultrasound exposure than the control agent. In a recent study, a new theranostic peptide platform (Cy5.5-iRGDC-Pt(IV)) could not only demonstrate an ideal tumor imaging effect but also induce tumor-specific apoptosis resulting in evident tumor suppression by conjugating a fluorescent dye and a cisplatin prodrug on each terminus of cyclic iRGD for simultaneous cancer-targeted imaging and therapy. In addition, the new platform had negligible systemic toxicity [34]. This area is an up-and-coming research hotspot and must be investigated thoroughly.

Previous studies and findings suggested that the iRGD peptide is a potential tumor target for more sensitive visualization and more effective gene therapy.

## 5. iRGD for Cancer Therapy

The functional mechanism of iRGD is well recognized and illustrated, and its preclinical investigation has been carried out thoroughly. The following substantial studies for cancer therapy in vitro and in vivo regarding different cancers are summarized in detail.

### 5.1. Breast Cancer

Breast cancer is a leading cause of death among women [1]. To date, there are no ideal treatment options except surgery and chemotherapy; thus, more effective and noninvasive treatment is urgently needed. Since the development of the iRGD peptide, it has been widely and continuously used in the study of breast cancer therapy. Sugahara et al. [13] investigated the efficacy of iRGD-Abraxane in a tumor model using the BT474 human breast cancer cell line. After intravenous injection, iRGD-Abraxane accumulated in the tumor 11-fold more than nontargeted Abraxane and approximately 4-fold more than CRGDC-Abraxane. In addition, significant tumor growth suppression was observed in vivo [13, 14, 34]. In 2013, Liu et al. [35] devised a new strategy to improve anticancer efficiency by conjugating a Dox-loaded crosslinked multilamellar liposomal vesicle (cMLV) to the iRGD peptide. The iRGD peptide facilitated and increased the binding ability and cellular uptake of cMLV in breast cancer cells. They also found that iRGD-conjugated cMLVs (iRGD-cMLVs) delivered into cells were regulated by the clathrin-mediated pathway. These findings suggest that the iRGD peptide can overcome the transport limitation of the targeted payload into the tumor parenchyma and establish tissue-penetrating anticancer drug delivery. The molecular mechanism by which iRGD exerts high-efficiency tissue penetration may be related to the vascular permeabilization induced by the CendR function of iRGD [13].

Another study by Cun et al. [36] developed a novel and scalable complex (iRGD-DOX-AuNPs-GNPs) and a tumor-microenvironment-responsive multistage system (DOX-AuNPs-GNPs) that was pH-sensitive. The data showed that coadministration of iRGD with DOX-AuNPs-GNPs increased cellular uptake, facilitated apoptosis in vitro, and exhibited higher penetration and accumulation resulting in the best antitumor efficiency in 4T1 tumor mice in vivo. The results may be due to the interaction between *α*v*β*3 and NRP-1 receptor overexpression in 4T1 cells. To potentiate chemotherapy, loaded liposomes modified by nRGD (nRGD-Lipo-Dox) were developed by covalently conjugating the alanine-alanine asparagine “tail” residues to the cyclic tumor-homing peptide iRGD in the 4T1 breast cancer mouse model; nRGD-Lipo-Dox showed a prominent antitumor effect by penetrating and accumulating in the tumor [37]. The use of doxorubicin-loaded low-temperature-sensitive liposomes (LTSL-DOX) modified by iRGD (iRGD-LTSL-DOX) was reported by Deng et al. [38]. iRGD-LTSL-DOX can specifically bind to *ανβ*3 on breast cancer cells and release encapsulated DOX under special conditions. In vivo, the results showed that DOX released and rapidly penetrated into tumor tissues after high-intensity focused ultrasound (HIFU) in the 4T1 breast tumor models, leading to enhancement of the drug's anticancer efficacy. Therefore, they concluded that iRGD penetration and the HIFU thermal effect jointly enhanced drug delivery.

iRGD-modified core-shell nanocapsules (iRGD-NCs) loaded with paclitaxel (PTX) were prepared by Jin et al. [39] The nanocapsules had some superior properties, such as increased drug loading, higher drug accumulation in tumors, higher cytotoxicity against cancer cells, longer circulation effects, and significant anticancer effects. First, iRGD-NCs showed a superior anticancer ability because iRGD increased NC accumulation and penetration in tumors. Second, iRGD significantly improved the pharmacokinetics of PTX, prolonged drug circulation in blood, and increased drug bioavailability.

In an in vivo study, Chen et al. [40] developed iRGD-CDD by conjugating Bit1 CDD to iRGD, which resulted in the compound possessing unique tumor-penetrating and cell-internalizing properties. In orthotopic implantations of MCFA-10CA1a and 4T1 breast cancer cells into mice, iRGD-CDD spread extensively when intratumorally injected and inhibited tumor growth significantly, leading to an average reduction of 77% in tumor volume and eradication of some tumors. They speculated that Bit1 and Bit1-CDD regulated the activities of the antiapoptotic and oncogenic transducing-like enhancer of split (TLE) proteins to mediate cell death. In addition, CendR peptides may deliver CDD into the cytoplasm and take part in protein transduction [40].

A cysteine residue was added to the iRGD peptide (Cys-iRGD) to prolong the cytoplasmic half-life of iRGD compared to that of parental iRGD, as reported by Pang et al. [41] Cys-iRGD accumulation in the tumor was more robust than that of parental iRGD. A new composition (Cys-X-iRGD) was synthesized by increasing the Cys-iRGD via insertion of a GGSGGSGG linker between them. The results demonstrated that Cys-X-iRGD induced more trastuzumab accumulation outside tumor blood vessels significantly than the control agent in 4T1 breast cancer. In addition, this function was regulated by covalent binding of iRGD to plasma albumin through a specific disulfide bond. Some polypeptide hormones modified by iRGD can also change the biological behavior of breast cancer cells and enhance targeted anticancer effects [42]. iRGD conjugated to thymosin alpha 1 (T*α*1-iRGD) showed higher binding ability to breast cancer cells and significantly inhibited MCF-7 cell growth. In addition, T*α*1-iRGD can enhance anticancer drug efficacy by increasing cell penetration and tumor accumulation. These findings may be related to the preservation of anticancer immunomodulatory activity of T*α*1 by enhancing the proliferation of spleen lymphocytes in mouse models. In addition, T*α*1-iRGD also considerably induced MCF-7 cell apoptosis, which might be related to the superior effect of T*α*1 on upregulating BCL2-associated X protein (Bax), caspase 9 expression, etc. [42]. The authors also produced a new product (TP5-iRGD) by fusing iRGD with the C-terminus of thymopentin (TP5), and it had the same anticancer effect in vitro and in vivo [43].

Kotamraju et al. [44] reported that FAM-X-C(iRGD)REKA extravasated into tumors interior from the blood vessels; however, the CREKA peptide alone homed only to the tumor vasculature in mice bearing MCF10CA1A human breast cancer xenograft tumors. Their findings imply that penetration into a tumor's interior is an internal function of iRGD.

Ma et al. [45] synthesized pH-sensitive fluorocarbon functionalized nanoparticles (SFNs) conjugated to the tumor-penetrating peptide iRGD. SFNs not only overcame their inherent instability but also facilitated dramatic tumor accumulation and penetration in an orthotopic breast cancer mouse model in vivo, resulting in a synergistic effect between iRGD and SFNs and a consequent increase in tumor necrosis or apoptosis, reduction in tumor angiogenesis, and suppression of Ki-67-positive tumor cell proliferation.

In vivo, induction of an antitumor immune response can also be a strategy for breast cancer therapy [46]. Deng et al. [46] found that anticancer drug accumulation in tumors was greatly increased when nanostructured lipid carriers (NLCs) coadministered with iRGD led to significant tumor growth suppression and an increase in immunogenic cell death. Therefore, the antitumor immune response of chemotherapy greatly benefits anticancer efficacy and renders a promising application for tumor therapy.

### 5.2. Lung Cancer

Numerous anticancer drugs or therapy modalities with iRGD targeting have been applied in lung cancers, including human non-small-cell lung cancer (NSCLC) and Lewis lung carcinoma in animal models. In 2012, Song et al. [47] explored the effect of methoxy poly(ethylene glycol)-block-poly(L-glutamic acid) (mPEG-b-PLG) loaded with cis-diamminedichloroplatinum (cisplatin, CDDP) in combination with iRGD for the treatment of NSCLC. In vivo, mPEG-b-PLG-loaded CDDP coadministered with iRGD exhibited elevated antitumor efficacy, resulting in the reduction of tumor volume and a prolonged survival time by over 30%, which was associated with the reduced toxicity, increased drug concentration in tumor, lower drug dose, and fewer side effects when CDDP was loaded with iRGD. Zhang et al. [48] improved anticancer efficacy of gemcitabine by coadministering it with the iRGD peptide in A549 xenograft mouse models. In vivo, tumor growth slowed and tumor weight decreased with gemcitabine+iRGD. The tumor growth inhibition rate of gemcitabine+iRGD was 86.9%, which was lower than that in the iRGD and gemcitabine groups. In addition, gemcitabine coadministered with iRGD inhibited cell proliferation and induced apoptosis in a human NSCLC-derived A549 cell line. Another study focused on coadministering cetuximab with iRGD in a murine model of human NSCLC [49]. Here, the data showed that cetuximab combined with iRGD could enhance tumor penetration and drug accumulation in tumor tissues, particularly at 3 and 9 h after cetuximab and iRGD administration. Because of the abundant overexpression of *ανβ*3, *ανβ*5, and NRP-1 in NSCLC cells, the binding and interaction of iRGD with these proteins facilitated the enhancement of anticancer drug effect [48]. Puig-Saus et al. [50] genetically inserted the iRGD peptide in the fiber C-terminus of an oncolytic tumor-retargeted adenovirus called ICOVIR15K to enhance its tumor penetration. The results showed that insertion of iRGD could increase tumor transduction and early adenovirus dissemination through the tumor and enhance anticancer efficacy in lung cancer. Lao et al. [51] revealed that thymosin alpha 1 (T*α*1) modified by the iRGD peptide inhibited cell proliferation and enhanced the specificity and potency of T*α*1 to the human lung cancer cell line H460. It was reported that T*α*1 was able to elevate the expression of histocompatibility complex class I surface molecules and tumor antigens in tumor cells, which indicated the potential of T*α*1 for enhanced anticancer activity [52]. In an in vitro experiment with Lewis lung carcinoma (LLC) cells, Solomon et al. [53] developed a tumor spheroid culture model and found that the iRGD peptide coadministered with unmodified liposomes presented higher accumulation and penetration features leading to higher anticancer activity. The authors summarize the following plausible explanations. First, the surface charge of the formulation may contribute to the high cell-binding ability to the liposomes. Second, the micelles were very small in size, which may be responsible for the higher penetration ability of the DOTAP-liposomes. Third, there might have been a significant loss in the membrane integrity of the cells of the spheroid.

Another difficult problem is drug resistance. The findings by Shen et al. [54] sought to overcome this difficulty. In their study, iPTPNs (iRGD-conjugated D-*α*-tocopheryl polyethylene glycol 1000 succinate mediated codelivery of paclitaxel and survivin shRNA) were formed, which simultaneously exerted an enhanced permeability and retention effect and an iRGD-mediated active-targeting effect. iPTPNs significantly enhanced the accumulation of PTX and survivin shRNA (shSur) and facilitated cancer cell apoptosis in tumors. The in vivo anticancer efficacy showed that the tumor volume of the iPTPN group (10 mg/kg) decreased dramatically. Therefore, iRGD-mediated PTX and shSur codelivery had the potential for lung cancer resistance reversal.

Some cytokines are also involved in tumor suppression and the selective induction of apoptosis in many human cancer cells, and some cytokines can exhibit enhanced function with the assistance of iRGD. A novel recombinant protein (IL-24-iRGD) was designed by fusing the C-terminal domain of interleukin-24 to the iRGD peptide. The results showed that IL-24-iRGD induced apoptosis and inhibited tumor growth to a significantly greater extent, which had a higher tumor growth inhibition rate (59.1%) than the control treatment (26.2%) [55].

### 5.3. Prostate Cancer

Prostate cancer is one of the leading causes of death in men, and distant metastasis is frequent in prostate cancer patients [1]. In recent years, many studies have focused on prostate cancer treatment. DOX coinjected with iRGD significantly enhanced penetration and accumulation more than 7-fold in orthotopic 22Rv1 tumors compared to DOX given alone. The efficacy of combination therapy was three times greater than DOX administration alone. A combination of 3 mg/kg DOX and iRGD showed a maximum benefit of DOX activity by inducing tumor growth inhibition based on stronger TUNEL staining, a sign of cell death [14]. De et al. [56] developed a new peptide with higher bioactivity named amphipathic tail-anchoring peptide-iRGD-M8 (ATAP-iRGD-M8), which improved stability and aqueous solubility in cultured cancer cells without conferring cytotoxicity. Because this peptide had a longer half-life in blood circulation due to degradation protection, improved solubility in physiological saline solutions, and the advantage of ATAP, it induced apoptosis independently of Bcl-2 family proteins. In addition, their findings showed significant tumor growth inhibition with intravenous injection of ATAP-iRGD-M8 [56]. Peng et al. [57] reported that a monolayer (2D) and multilayer (3D) of DU-145 prostate cancer cells treated with tumor-penetrating peptide conjugates (P-DOX-PLGLAG-iRGD) accumulated more DOX than control-treated cells did. Meanwhile, P-DOX-PLGLAG-iRGD showed the best penetration ability in 3D multicellular DU-145 tumor cell spheroids. Multifunctional porous silicon (PSi) nanoparticles modified with iRGD enhanced the uptake of nanoparticles by tumor cells in ectopic PC3-MM2 mouse xenograft models. Here, PSi nanoparticles were delivered and distributed in the tumor, leading to efficient tumor growth inhibition with nanoparticle-loaded sorafenib compared to that of free drug due to the fast release of sorafenib from the PSi matrix into the blood after intravenous injection and its limited dissolution rate in blood circulation [58].

Theranostic nanoparticles have also been applied in the treatment of prostate cancer. A polymer nanoparticle (IO@PNP) was synthesized by Herranz-Blanco et al. [59]. IO@PNPs were largely internalized by endothelial (EA.hy926) and metastatic cancer (PC3MM2) cell lines. The internalization rate of iRGD-conjugated IO@PNPs into EA.hy926 cells was moderately increased, but no enhancement was observed with PC3MM2 cells. Poly(ethylene glycol)-*block*-poly(histidine) with pH-responsive and proton-sponge characteristics facilitated rapid lysosomal escape. In addition, IO@PNP-doxorubicin and iRGD showed both intracellular lysosomal escape and efficient transfer of doxorubicin to the nuclei of cells. In summary, the IO@PNP-doxorubicin with the iRGD modification enhanced the anticancer efficacy of DOX by improving the intracellular delivery of DOX encapsulated in IO@PNPs.

### 5.4. Melanoma

Melanoma is a highly malignant tumor with a high risk of metastasis. Therefore, targeting melanoma therapy has attracted broad attention. Su et al. [60] developed a multifunctional nanogel for melanoma targeting using DOX-encapsulated iRGD-decorated nanogels (NGs), which facilitate drug release from B16 tumor cells because of the maintenance of their thermo- and pH-responsiveness features. The intracellular uptake of DOX-NGs was increased remarkably via iRGD modification in B16 tumor cells. NGs with a pH-responsive function showed controlled DOX release in deep tumors and much fewer side effects from DOX, leading to maximal anticancer efficacy in vivo. A new sterically stabilized liposome (SSL) with DOX-loaded and iRGD-modified (iRGD-SSL-DOX) was applied to B16-F10 cells in vitro and to tumor-bearing mice in vivo because *α*v integrin receptor and NRP-1 were also overexpressed in B16-F10 cells [61]. All the in vitro and in vivo results showed that tumor-targeting and tumor-penetrating activities of SSL modified with iRGD were much more enhanced than those of the control treatment. The antitumor efficiency against melanoma tumors also increased significantly, which was reflected by the substantial inhibition of tumor growth and reduction in tumor size [62]. Another similar study was performed using a novel liposome modified with iRGD in B16-F10 melanoma, which contained the conjugated linoleic acid-paclitaxel (iRGD-SSL-CLA-PTX). Here, cellular uptake of the drug and CLA-PTX levels after iRGD-SSL-CLA-PTX treatment increased at different time points. The in vivo experiments demonstrated that iRGD-SSL-CLA-PTX greatly suppressed the growth of B16-F10 tumors [63] because the benefits in increasing drug exposure were improved through ligand-modified delivery systems in vitro and the enhanced permeability and retention effect (PRE) [64]. Interestingly, Deng et al. [65] developed a new anticancer synthesis-oligomeric hyaluronic acid-liposome (HA-Lip) for melanoma. When coadministered with iRGD, HA-Lip could penetrate into the tumor more efficiently because of the enhanced internalization through receptor-mediated endocytosis on tumor cell surfaces. Meanwhile, in the in vivo test, DOX-loaded HA-Lip coadministered with iRGD showed a considerably improved anticancer effect on melanoma. Thymosin alpha 1 (T*α*1) modified by the iRGD peptide also inhibited B16F10 cell proliferation and enhanced the targeting and binding affinity in vitro [51].

### 5.5. Gastric Cancer

Gastric cancer is a common malignant tumor of the digestive system that has a poor prognosis. New molecular-targeted therapies may be a potential strategy for gastric cancers. Thus, gastric cancer-targeted therapy has prevailed since 2015. Sha et al. [66] demonstrated that PTX combined with anti-EGFR-iRGD showed a higher attachment and binding affinity in three human gastric cancer cell lines and inhibited tumor growth in vivo with an approximate tumor volume reduction of 46.66% compared with the control. The improved anti-EGFR-iRGD has significant advantages in solid tumor therapy, especially for the treatment of gastric cancer with various drugs, including DOX, bevacizumab, and PTX, and this effect may be related to the delay of macrophage-mediated drug clearance, which facilitated persistent circulation and enhanced drug delivery into tumors more efficiently [67]. KLA-iRGD, another functional recombinant protein, was constructed [68]. KLA is a peptide that can disrupt mitochondrial membranes, resulting in cell death. iRGD can facilitate KLA function in cell death. KLA-iRGD inhibits tumor growth via activation of the receptor neuropilin-1 and subsequent internalization activity. KLA-iRGD spreads rapidly and extensively in the gastric tumor mass. The recombinant KLA-iRGD protein considerably reduces tumor size and volume, which indicates that it has a higher anticancer efficacy in mice in vivo.

iRGD functionalization potentiated intraperitoneal PTX-loaded pH-sensitive poly(oligoethylene glycol methacrylate)-poly(2-(diisopropylamino)ethyl methacrylate)-polymersomes (POEGMA-PDPA-PS) tumor selectivity and anticancer activity in gastric cancer mouse models. The anticancer efficacy of iRGD-PS-PTX was enhanced greatly compared to that of untargeted PS. Through their study, Dai et al. revealed that the CendR peptide RPARPAR is able to recruit polymersomes to NRP-1 and target polymersomes to cells positive for NRP-1 expression, and PTX-polymersomes targeting CendR peptides showed selective cytotoxicity for NRP-1-positive cells [62]. In addition, under physiological pH conditions, iRGD-polymersomes rapidly disassembled and released PTX after cellular internalization, which may contribute to the increased chemotherapy efficacy [69, 70]. In 2017, some novel targeting methods and treatment modalities were investigated. sTRAIL-iRGD (soluble tumor necrosis factor-related apoptosis-inducing ligand-iRGD), a recombinant protein consisting of sTRAIL fused to an iRGD peptide, was synthesized by Huang et al. [71]. During both in vitro and in vivo studies, sTRAIL-iRGD combined with PTX had significant antitumor efficacy. Repeated treatment with sTRAIL-iRGD-PTX inhibited tumor growth and reduced tumor volume in vivo. sTRAIL was cleaved proteolytically from TRIAIL and retained the characteristics along with the positive effects of iRGD, including apoptosis induction in transformed cancer cells, no negative side effects to the host, and significant anticancer activity.

iRGD could also potentiate the anticancer effect of 5-fluorouracil (5-FU) on gastric cancer cells through NRP1. In vitro, cells treated with 5-FU + iRGD weakened cell viability, proliferation, migration, and invasion. In vivo, the anticancer efficacy of 5-FU was dramatically enhanced with iRGD treatment, resulting in slower tumor growth, but the distinct mechanism by which iRGD regulates 5-FU is still being investigated [72].

Cell immunotherapy is another potential therapy, but it is limited by the poor infiltration of activated lymphocytes into tumors. However, exciting progress in cell immunotherapy has already begun to become integral to cancer treatment strategies [73]. iRGD-modified T cells were able to penetrate into the core of the three-dimensional multicellular sphere when a time-efficient platform that links iRGD to the T cell surface was applied. By contrast, T cells alone could not penetrate the spheres. Meanwhile, iRGD modification could increase T cell numbers in the tumor parenchyma up to 10 times in different tumor models in vivo. Moreover, the iRGD modification had a synergistic effect with programmed death-1 (PD-1) disruption in enhancing the anticancer effect and extending the survival time in mouse models [73].

### 5.6. Liver Cancer

As early as 2011, studies on hepatocellular carcinoma-targeted therapy with the iRGD peptide began. Poly(*ε*-caprolactone)-b-poly(N-vinylpyrrolidone) (PCL-b-PVP) copolymers with different PVP block lengths and PTX-loaded nanoparticles that contained 15% drugs and had more than 90% encapsulation efficiency were developed. iRGD-PTX-PCL-PVP showed superior cytotoxicity and cellular uptake of the particles, which indicated that the iRGD peptide contributes to the interaction of the nanoparticles with tumor cells through NRP-1 overexpression on the cell surface. iRGD-PTX-PCL-PVP facilitated the accumulation of nanoparticles at the tumor site because the small nanoparticles (<50 nm) can easily penetrate through the endothelial wall of hepatic cells, leading to enhanced liver uptake [74]. The permeability of the nanoparticles was greatly improved, and the best anticancer efficacy was exhibited in the H22 tumor-bearing mouse models. This effect might be related to the circulatory nanoparticles in vivo, which were trapped in the tumor via the EPR effect or active affinity [75]. Endostatin is a 20 kDa C-terminal fragment, 183 amino acid residues in length, that is derived from collagen XVIII [76] and has been used in cancer treatment for its anticancer activity. iRGD-modified endostatin became much more efficient as a liver cancer therapy. The results indicated that iRGD-modified endostatin inhibited cell proliferation and migration and enhanced endostatin distribution largely in tumors. The antitumor activity and liver cancer growth suppression are due to the neovascularization block by endostatin via iRGD binding to *α*_V_*β* integrins [77]. M-SAL-iRGD for both liver cancer cells and cancer stem cells (CSCs) salinomycin delivery was synthesized by Mao et al. [78]. M-SAL-iRGD showed great superiority with a small size (10 nm) and higher drug encapsulation efficacy (more than 90%). M-SAL-iRGD enhanced cytotoxicity, greatly targeting M-SAL and salinomycin in both liver cancer cells and CSCs. M-SAL-iRGD showed a greater tissue distribution and anticancer efficacy in liver cancer-bearing mice. The anticancer mechanism of the M-SAL-iRGD is elaborated as follows. First, M-SAL-iRGD accumulates in tumors because of the increasing EPR effect. Second, M-SAL-iRGD binds to *α*_V_*β* integrin and is subsequently proteolytically cleaved into the M-SAL-CRGDK/R motif, which has a higher affinity for NRP-1. Third, NRP-1 binding activates tissue penetration. Finally, after internalization, salinomycin is released from M-SAL-iRGD rapidly in the cytoplasm and kills hepatocellular carcinoma cells and CSCs [78]. iRGD-modified lipid-polymer hybrid NPs with a shell core synthesized for DOX and sorafenib (SOR) codelivery (DOX+SOR/iRGD NPs) were observed by Zhang et al. [79]. DOX+SOR/iRGD NPs showed an increasing synergistic cytotoxicity in cell proliferation and an apoptosis internalization rate in HepG2 cells due to many advantages, such as the synergistic cytotoxicity of drugs, sustainable drug release, enhanced cellular internalization, longer circulation time, and enhanced tumor accumulation. More importantly, DOX+SOR/iRGD NPs showed optimal anticancer activity and apoptosis efficacy in vitro and in vivo mediated by the iRGD interaction with *α*_V_*β* integrin. Schmithals et al. [80] reported that the tumor penetrability of sorafenib and doxorubicin could be considerably enhanced in hepatocellular carcinoma with iRGD through a synergistic effect. They also found that iRGD potentiated the efficiency of drug delivery (approximately threefold) to a specific site in HCC-bearing mouse models. Similar results were observed in a study by Wang et al. [81]. iRGD−PEG-PLA-loaded vandetanib had greatly improved therapeutic efficacy, leading to nearly 60% tumor growth suppression compared to 20 mg/kg vandetanib administration alone. All the above studies suggested that the anticancer effect was enhanced by the interaction of iRGD with *α*_V_*β* integrin expressed on liver cancer cells.

### 5.7. Pancreatic Cancer

The pancreas is a retroperitoneal organ adjacent to the celiac plexus [82]. Pancreatic cancer is a highly malignant tumor that invades the celiac plexus and metastasizes to other organs [83]. Currently, drug treatments for pancreatic cancer are unsatisfactory. The advent of the iRGD peptide is good for pancreatic cancers because conjugated iRGD can be used directly due to the presence of *α*_V_*β* integrin on the pancreatic cancer cell surface. Targeted therapies for pancreatic cancer have made great strides.

In 2009, a fluorescein-labeled iRGD (FAM-iRGD) was synthesized and added to pancreatic cancer cells (MIA PaCa-2) [13]. FAM-iRGD accumulated in or around tumor vessels and tissues and showed strong fluorescence compared to that in normal tissues, which indicated that FAM-iRGD exhibits strong homing and binding affinity in tumors. For the in vivo tests, FAM-iRGD accumulated and is retained within the tumor because of the increasing permeability in tumor vessels and strong positive expression of neuropilin-1 in tumor cells, which suggests that vascular permeabilization is involved in the molecular mechanism of iRGD-mediated rapid tumor tissue penetration. In pancreatic cancer mouse models with NRP-1 overexpression (BxPC-3 and MIA PaCa-2), gemcitabine coadministered with iRGD showed enhanced tumor penetration and anticancer ability in comparison with gemcitabine alone as confirmed by pancreatic cancer models. These models also revealed that the iRGD effect largely depends on the level of NRP-1 in the tumor, which indicated that the iRGD peptide-mediated effect may be exploitable in some pancreatic cancer patients with high NRP1 expression [84]. Liu et al. [85] developed a multifunctional mesoporous silica nanoparticle (MSNP) designated as a “silicasome”, which contains a nonsupported lipid bilayer (LB), to improve the efficacy of pancreatic ductal adenocarcinoma (PDAC) chemotherapy. Silicasome showed a significantly increased drug-loading ability, improved stability in vessels, and reduced drug loss during circulation. Through research and analysis, the transcytosis pathway, EPR effect, nutritional transport pathways, and vascular growth factors may be involved in the molecular mechanism [86, 87]. Another iRGD-nanocage targeting system for pancreatic cancer was described [88]. The iRGD domain was connected to the C-terminal region of heat shock protein (HSP) to form an iRGD-nanocage. An L30-iRGD-nanocage with 30 amino acid linkers exhibited superior binding affinity to pancreatic cancer cells (AsPC-1) and increased cellular uptake, which was related to the length of the linker between the nanocages and the iRGD domain. In addition, OSU03012 (a hydrophobic anticancer drug)-loaded iRGD-nanocages induced AsPC-1 cell death more efficiently in vitro by activating the caspase cascade because of the presence of the iRGD domain; the internalization was accelerated by interactions between the iRGD domain and NRP-1 on the surface of pancreatic cancer cells [89] and by the bystander effect [14]. In a new report in 2017, Tsang et al. [90] found that tumor-targeting peptides (iRGD and cRGD) coupled to U1 adaptors with fluorescence presented targeted tumor localization and highly potent anticancer efficacy (>90%) by targeting two oncogenes (KRAS and MYC) in pancreatic cancer in vivo. Because U1 Adaptor is a new generation gene silencing technology that may serve as a new therapeutic modality for targeting any oncogene [91], U1 Adaptors were translated to target KRAS and MYC, and the adaptors strongly inhibit pancreatic cancer cell proliferation. The iRGD-tumor-penetrating nanocomplexes (iRGD-TPNs) with polyethylene glycol (PEG)-peptide conjugates delivering anti-KRAS siRNA to tumor sites significantly delayed tumor growth in murine models. The significance of iRGD-TPNs is their ability to not only overcome physical barriers to therapy but also achieve knockdown of the gold standard genetic target by leveraging the stroma [92].

Finally, oncolytic adenovirus modified by iRGD by insertion of an iRGD peptide sequence in the fiber C-terminus of ICOVIR15K could also enhance pancreatic cancer (MIA PaCa-2) cell transduction, intratumoral spread, and anticancer efficacy with a great reduction of the tumor volume. The reasonable explanations are as follows: First, the insertion of the iRGD peptide in the fiber C-terminus of ICOVIR15K boosted the binding and internalization ability of the virus in cells that highly expressed NRP-1. Second, the insertion of iRGD and RPARPAR peptides did not damage the basal characteristics of adenovirus update through integrin receptor-mediated endocytosis. Third, the phenotype of iRGD was additive to the KKTK-to-RGDK fiber shaft modification, which was proven to enhance tumor transduction and anticancer activity [50, 93].

### 5.8. Glioma

Many functional peptides could enhance the binding affinity to gliomas. MT1-AF7p, a newly identified peptide, showed a prime affinity for membrane type-1 matrix metalloproteinase (MT1-MMP), which is an ideal antiglioblastoma target [94]. Gu et al. developed MT1-AF7p-conjugated nanoparticles (NPs) by employing MT1-MMP modifying paclitaxel-loaded PEG-PLA nanoparticles (MT1-NP-PTX). iRGD coadministration with these nanoparticles significantly enhanced its penetration across the blood-brain tumor barrier (BTB) and increased its accumulation in glioma parenchyma. It also significantly improved the antiglioma effect in mice bearing C6 glioma tumors. This enhanced effect was deemed to benefit from the transcytosis mediated by MT1-AF7p and iRGD-facilitated NP extravasation and tumor penetration [95]. More importantly, the MT1-AF7p peptide may contribute to increasing NP penetration into the glioma itself [87]. Wang et al. [96] produced an iRGD-PPCD conjugate to investigate its effect on glioma. The results indicated that the iRGD-mediated PPCD delivery system possessed a higher penetrating ability and superiority because the enhanced cellular internalization of the conjugate was activated by *α*_V_*β* integrins on the C6 cell surface. After systemic administration in vivo, iRGD-mediated PPCD demonstrated potent penetration ability, higher accumulation in tumors leading to tumor vascular density, and a reduction in the average vascular diameter. The underlying mechanisms for the enhancement of tumor accumulation and penetration are summarized as follows: (1) the conjugate is long-circulating and has an applicable size; (2) iRGD peptides coupled to PPCD activate tumor-targeted properties to further improve the accumulation; and (3) the penetration of cells and tissues for iRGD-mediated PPCD is activated by proteolytic cleavage to expose the cryptic CendR, which binds to NRP-1 on the cell surface [87]. In a recent study, chitosan surface-modified poly(lactide-coglycolide) nanoparticles (PLGA/CS NPs) loaded with carmustine (BCNU) and its sensitizer (O^6^-benzylguanine, BG) were prepared [97]. iRGD-modified NPs or iRGD coadministration with NPs showed significant enhancement of tumor penetration, accumulation, and antitumor activity. In addition, the median survival time of iRGD NPs or iRGD+NPs was prolonged in mice bearing F98 gliomas. The potential mechanisms of the iRGD-mediated PLGA/CS NP system with enhanced efficacy are similar to those identified in the study by Wang et al. [96]. Another explanation is also significant: the BG was released from the CS shell of NPs and then depleted O^6^-methylguanine-DNA-methyltransferase, so the tumor cell sensitivity to BCNU increased, which was released later from the PLGA core [97].

### 5.9. Cervical Cancer

The applications of iRGD in cervical cancer therapy have also been investigated in recent years. A-PTX-SF-NPs were synthesized using PTX-SF-NPs joined to anti-EGFR-iRGD because it was a simple method and showed better anticancer activity. Furthermore, there was a high number of carboxyl groups on the PTX-SF-NP surface, which could combine with the recombinant protein through carbodiimide [98]. A-PTX-SF-NPs had a small size, penetrated the tumor tissues easily, and showed increased cytotoxicity towards the tumor cells. In HeLa tumor-bearing nude mice, A-PTX-SF-NPs exhibited better tumor-targeting and anticancer efficacy in vivo, and an EPR effect was obtained [99].

Mesoporous silica nanoparticles (MSNs) conjugated with iRGD, a novel tumor-targeting delivery system loaded with combretastatin A4 (CA4) and doxorubicin (DOX), were used to improve antiangiogenesis activity and chemotherapy efficacy [100]. MSNs-iRGD target *α*_2_*β*_3_ integrin receptors expressed in cervical cancer cells and vasculature cells. MSNs-iRGD loaded with CA4 and DOX could accumulate at the targeted tumor site due to the prolonged blood circulation, which resulted in better targeting to the vessel wall. CA4 is first released to disrupt the tumor vasculature and reduce the tumor blood supply; then the impaired tumor vasculatures would promote the penetration of the drug delivery system. Finally, the chemotherapy drug DOX was released into the circulation to promote apoptosis of cancer cells. Thus, the anticancer efficacy was enhanced significantly in vivo.

### 5.10. Colorectal Cancer

The effect of polymersomes, polysaccharide nanoparticles, and anticancer drugs with iRGD was also observed in colorectal cancer therapy through a CendR motif binding to NRP-1 [69, 101–103]. Due to the internalization of iRGD and NPR-1, tumor cell penetration and tissue accumulation were enhanced. Intraperitoneally administered iRGD-polymersomes showed higher tumor-specific accumulation and penetration in mice bearing colon cancer tumors comprising CT26 cells. iRGD-polymersomes loaded with PTX potentiated the tumor growth and metastasis inhibition effects in vivo, and the results indicated that the combination of direct penetration and circulation-mediated homing with iRGD was involved, which facilitated payload delivery to the targeted tumor sites [69]. Sugahara et al. [102] investigated the function of iRGD in improving tumor-targeting penetration of intraperitoneal compounds and enhancing intraperitoneal chemotherapy (IPC) in mice bearing human colon cancer tumors. The data showed that intratumoral entry of dextran and DOX was enhanced by approximately 300% and 250%, respectively, after intraperitoneal coinjection with iRGD. Meanwhile, bulky peritoneal tumor growth was suppressed, and systemic drug toxicity decreased when using intraperitoneal iRGD/doxorubicin combination therapy depending on the CendR-mediated transtumoral bulk transport system, which implied that intraperitoneal iRGD combined with IPC could be a simple yet effective approach to treating colorectal cancer and peritoneal carcinomatosis due to higher intratumoral drug accumulation even in the presence of ascites. Doxorubicin-loaded polysaccharide nanoparticles (Dex-SA-DOX-CDDP NPs) coadministered with iRGD can efficiently inhibit tumor growth in both subcutaneous transplantation colorectal carcinoma and primary colorectal carcinoma with CT26 murine colon carcinoma cell lines due to the prolonged circulation, enhanced tumor localization, and accumulation in the tumor via the EPR effect [86, 101].

Ma et al. [103] devised a new strategy (PEGylated camptothecin-loaded poly(lactic acid/glycolic acid) nanoparticles, iRGD-PEG-NPs) to enhance drug tumor accumulation and targeted delivery for colon cancer therapy with iRGD modification and a camptothecin (CPT) payload. They found that this new nanoparticle loaded with CPT had enhanced tumor accumulation, induced apoptosis, and efficiently downregulated* Bcl-2 *mRNA expression, which was deemed to promote apoptosis and inhibit tumor growth in orthotopic colon tumors in mice in vivo. The possible reasons for the improved anticancer efficacy may be the following: (1) NPs have an appropriate size with an average diameter less than 280 nm, which results in the optimally enhanced permeability and retention effect and the avoidance of uptake by the reticuloendothelial system or rapid renal clearance [104]. (2) iRGD-PEG-NPs enter cells via receptor-mediated endocytosis to enhance the cellular uptake efficiency of NPs and enhance the cytotoxicity of CPT-loaded NPs. (3) iRGD-PEG-NPs have excellent hemocompatibility, which facilitates chemotherapeutic efficacy.

### 5.11. Ovarian Cancer

The synergistic effect of ovarian cancer therapy with iRGD was less studied. The expression of *α*v integrins and NRP-1 on the surface of ovarian cancer cell facilitates the function of iRGD. Fluorescein-iRGD (FAM-iRGD) efficiently penetrated IGROV-1 xenograft tumors with 30 mm thickness, which indicated promising prospects for iRGD in delivering drugs into human ovarian tumors [102]. An urgent challenge for ovarian cancer therapy is to overcome multidrug resistance (MDR). Zhang et al. [105] developed a new codelivery system (PTX+TET/iRGD LPNs) with iRGD-modified lipid-polymer hybrid nanosystems (LPNs) coupled to paclitaxel (PTX) and tetrandrine (TET) at a precise ratio (1/1 molar ratio) to overcome MDR. They found that the delivery system conferred higher anticancer drug load capacity, stable properties, and redox-sensitive drug release profiles. In their study, they designed a coloaded LPN profile to release drugs in sequential order. TET was released first in cells, and then PTX was released in the cytoplasm. This ingenious design improves the anticancer efficacy. Owing to these superior properties, when treated with PTX+TET/iRGD LPNs, A2780 ovarian cancer cells showed much more PTX accumulation. In addition, PTX+TET/iRGD LPNs exerted significant cytotoxicity to A2780/PTX cells, enhanced reactive oxygen species (ROS) production, and induced apoptosis. These findings demonstrate the important role of iRGD in ovarian cancer therapy.

## 6. iRGD in Inhibiting Tumor Metastasis

Tumor metastasis is another leading cause of death in cancer patients [1]. Therefore, many studies also focus on improving drug resistance and enhancing anticancer efficacy. The iRGD peptide itself had no effect on tumors or metastatic lesions; however, iRGD exerted its inhibitory function on tumor metastasis by delivering drugs to the tumor and by targeting integrins expressed on the cancer cell surface.

In 2015, Sugahara et al. [102] revealed that iRGD peptide coadministered with anticancer drugs via intraperitoneal injection could improve the therapeutic index and inhibit human peritoneal metastasis explants. In another study, Sugahara et al. [18] showed that the iRGD peptide inhibited cancer cell attachment to fibronectin and inhibited cancer cell migration in vitro through interactions with NRP-1. iRGD possessed chemorepulsive properties and inhibited prostate and pancreatic cancer metastasis in nude mouse models bearing GFP-PC-3 and LM-PmC tumors. Hamilton et al. [106] reported that iRGD-iron oxide nanoworms significantly inhibited breast cancer brain metastasis and significantly affected tumor progression in the early stages of metastasis. These findings were verified in two different models using MDA-MB-231 and 4T1 cell lines in vivo. The antimetastatic activity of iRGD may be regulated by NRP binding, which has been shown to inhibit tumor growth and metastasis [107]. In addition, iRGD peptide coated onto NPs extended the half-life of the peptide in the circulation, resulting in a prolonged time of activity at the target site [108]. Ni et al. [109] found that iRGD conjugated to PTX nanocrystallites (in the form of nanodots and nanoparticles) can endow nanocrystallites with a much higher drug-loading capacity, superior monodispersibility, and specific tumor-targeting ability, subsequently leading to cancer stem cell elimination and restricting breast cancer growth and metastasis in murine models. Li et al. reported that doxorubicin-loaded cisplatin crosslinked polysaccharide-based nanoparticles (Dex-SA-DOX-CDDP NPs) combined with iRGD can synergistically and efficiently suppress primary breast tumor growth and inhibit the metastasis of 4T1 murine orthotopic mammary carcinoma because of the prolonged circulation and enhanced tumor localization and accumulation of the anticancer drug in tumors via the EPR effect [101]. In 2016, Qifan et al. [110] showed that iRGD administration activated neuropilin-1 on the tumor cell surface and facilitated the internalization of the procytotoxic peptide (m(KLA)-iRGD) into 4T1 tumor cells. Then, apoptosis was rapidly triggered by m(KLA)-iRGD through the mitochondrial-induced apoptotic pathway and the death receptor pathway in NRP1+/*α*v*β*3/Cathepsin B+ tumor cells. Finally, after the m(KLA)-iRGD peptide was intravenously administered at an appropriate dose, lung metastasis was completely blocked. All these findings indicate the important functions of iRGD in inhibiting tumor metastasis.

## 7. Conclusion

In summary, the biofunctional iRGD peptide can facilitate tumor imaging as well as cancer and metastasis therapy through interactions with integrins expressed on the tumor cell surface; these intrinsic characteristics render it a promising prospect in tumor imaging and therapy. Because of the targeting features of iRGD, some imaging agents, polysomes, anticancer drugs, immune modulators, and cytokines can be modified with or conjugated to iRGD to penetrate tumors more deeply and effectively, leading to much more satisfactory imaging and enhanced antitumor efficacy. The function and effect of the iRGD peptide have been tested and confirmed in vitro and in vivo by an immense number of concrete studies. Therefore, the iRGD peptide serves as a potential and promising target for improving imaging and therapeutic efficacy in humans.

## Abbreviations

iRGD: Internalizing Arg-Gly-Asp peptide
NRP-1: Neuropilin-1
MRI: Magnetic resonance imaging
EGFR: Epidermal growth factor receptor
NP: Nanoparticle
QD: Quantum dot
MB: Microbubble
CCR2: C-C chemokine receptor type 2
DOX: Doxorubicin
HIFU: High-intensity focused ultrasound
CDD: Cell death domain
BCL-2: B-cell lymphoma 2
PTX: Paclitaxel
sTRAIL: Soluble tumor necrosis factor (TNF)-related apoptosis-inducing ligand (TRAIL)
iPTPNs: iRGD-conjugated D-*α*-tocopheryl polyethylene glycol 1000 succinate mediated codelivery of paclitaxel and survivin shRNA
M-SAL: Salinomycin-loaded DSPE-PEG2000 nanomicelles
DSPE-PEG2000: 1,2-Distearoyl-*sn*-glycero-3-phosphoethanolamine-*N*-[methoxypolyethylene glycol)-2000] (ammonium salt)
SOR: Sorafenib
PEG-PLA: Poly(ethylene glycol)-poly(lactic acid)
PPCD: PEGylated polyamidoamine (PAMAM) dendrimer-cis-aconityl-doxorubicin
PLGA/CS NPs: Chitosan surface-modified poly(lactide-co-glycolide) nanoparticles
PTX-SF-NPs: PTX-loaded silk fibroin nanoparticles
EPR: Enhanced permeability and retention
MDR: Multidrug resistance
Dex-SA-DOX-CDDP NPs: Succinic acid decorated dextran doxorubicin-loaded cisplatin (CDDP) crosslinked polysaccharide-based nanoparticles.
